# Treatment following myocardial infarction in patients with schizophrenia

**DOI:** 10.1371/journal.pone.0189289

**Published:** 2017-12-13

**Authors:** Rubina Attar, Martin Berg Johansen, Jan Brink Valentin, Jørgen Aagaard, Svend Eggert Jensen

**Affiliations:** 1 Department of Cardiology, Aalborg University Hospital, Aalborg, Denmark; 2 Department of Clinical Medicine, Aalborg University Hospital, Aalborg, Denmark; 3 Unit of Clinical Biostatistics, Aalborg University Hospital, Aalborg, Denmark; 4 Department of Psychiatry, Aalborg University Hospital, Aalborg, Denmark; SPAIN

## Abstract

**Background:**

A correlation between excess mortality from myocardial infarctions (MI) and schizophrenia has already been established. What remains unclear is whether the initial communication between the treating doctor and the corresponding patient contributes to this excess mortality.

**Aim:**

The aim of this study is to investigate whether a patient with schizophrenia receives the same offers for examination and treatment following a MI compared to a psychiatric healthy control (PHC).

**Methods:**

This cohort study includes patients diagnosed with schizophrenia at the time of their first MI (n = 47) in the years between 1995–2015 matched 1:2 to psychiatric healthy MI patients on gender, age and year of first MI. All existing hospital files for the 141 patients were thoroughly reviewed and the number of offered and accepted examinations and treatments were extracted for comparisons between the two groups.

**Results:**

In general patients with schizophrenia were less likely to be offered and accept examination and at the same time be offered and accept treatment as compared to PHCs (p<0.01). In addition, there was a statistical trend towards patients with schizophrenia being more likely to decline examination (p = 0.10) and decline treatment (p = 0.09) compared to PHCs, while being offered examination and being offered treatment both contributed statistically insignificantly to the overall discrepancy between the two patient groups.

**Conclusions:**

Being diagnosed with schizophrenia limits the treatment received following a first MI compared to PHCs. However, we are unable to pinpoint, whether Physician bias, patient’s unwillingness to receive health care or both contribute to the excess mortality seen in these comorbid patients.

## Introduction

An increasing amount of literature has confirmed a positive correlation between mental disorders and increased mortality [1,2], particularly in patients with schizophrenia [3–9]. The average age of death in patients with schizophrenia independent of self-harm has decreased over the past three decades while it has increased in the general population. This pattern results in an increased gap in life expectancy between the populations [10]. The excess mortality can be linked to an increased prevalence of cardiovascular diseases (CVD) in patients with schizophrenia [11–14]. In particular, a strong correlation between myocardial infarctions (MI) and schizophrenia has been established [2,15,16]. The increased prevalence of CVD can be explained by the unhealthy lifestyle of this population consisting of excessive smoking and drinking, an unbalanced diet together with the use of antipsychotic drugs which leaves the patient at greater risk of developing CVD [7,17,18].

**Fig 1:**
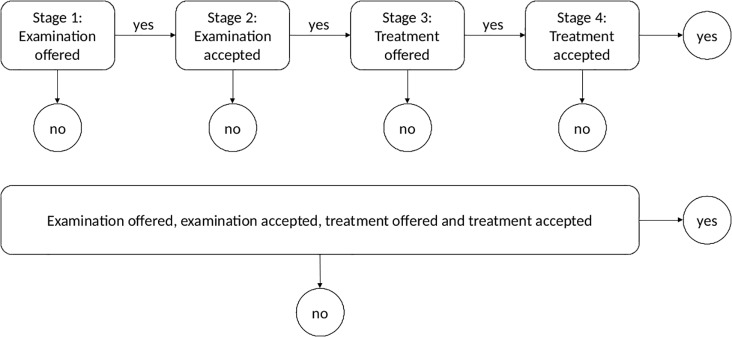
Above; illustrates the four stages in the pretreatment process used for the secondary subgroup analyses. In each of the four stages we investigate whether examination and treatment was offered by the doctor and accepted by the corresponding patient following a first MI. Patients were only considered susceptible to a stage if they completed the previous stage with positive outcome, otherwise they were removed from further analysis. Below; illustrates the pretreatment process used for the primary analysis, in which all stages are truncated into one.

It has already been established that patients with schizophrenia undergo fewer cardiac procedures compared to the general population [4,19,20]. However, it remains unclear whether these patients receive the same offers of examination and treatment by their doctor compared to the psychiatric healthy control (PHC) population. This study focuses on the four-stage process from initial admission following MI including offer of examination, accept of examination, offer of treatment and finally the accept of treatment which leads to the overall outcome of completing all four stages with positive outcome (Fig 1). Thus, the aim of this study is to investigate the number of patients who completes all four stages and accepts treatment in the final stage. Secondary analyses will cover the relative role played by the doctor and the patient following admission for MI.

## Methods

### Study design

This cohort study investigates received cardiac treatment in patients with schizophrenia compared to a PHC population through the process of reviewing the patients’ hospital files and analysing the initial communication between the doctor and patient after admission following MI.

### Data collection

All persons born in or permanently residing in Denmark are a part of the Danish Civil Registration System [21]. Each person is assigned a 10-digit “Central Person Register” (CPR) number, facilitating the unique linkage between different national registers, the local patients administrative system and the psychiatric patient database [22]. The different registers include information on gender, age and patient hospital files including date of hospitalisation, discharge diagnosis and procedures performed during the admission. The initial search for patients with an ICD-10 MI diagnosis (I21) including both ST elevation MI (STEMI) and non ST-elevation MI (NSTEMI) in the period of 1995–2015 resulted in a list of 12.902 people, all registered as residents of Northern Denmark. This list was cross-linked with an ICD-10 schizophrenia diagnosis (F20) resulting in a final list of 47 patients diagnosed with schizophrenia at the time of their first MI. Each of the 47 patients was assigned two PHC controls, matched on year of first MI, gender and age. All existing hospital files for the 141 patients were thoroughly reviewed and relevant data was collected in electronic case report forms. The data were analysed anonymously. The study was authorized by the Danish Data Protection Agency (2008-58-0028) and patient identification through registers ware approved by the Danish Health Data Authority (FSEID-00002258).

### Statistical analysis

Descriptive statistics are presented as either means and standard deviations or frequencies and percentages as appropriate. The descriptive data includes gender, age, BMI, smoking status, history of diabetes, hypercholesterolemia, hypertension and family predisposition for CVD. It is a standard procedure to include family predisposition during admission for CVD. The primary and secondary outcomes were dichotomous and included: if the treating physician offered examination and treatment to the patient and if the patient accepted the offer made. The secondary subgroup analyses only included susceptible patients, i.e. only patients who were offered examination were included in the subsequent analyses, and only patients who accepted examination were included in the analyses on treatment and so forth. All patients were included in the primary analysis testing how many patients actually completed all four stages with positive outcome (Fig 1). Finally, we compared what type of examination and treatment was offered. The outcomes and descriptive data were compared between the two groups of patients using a t-test for continuous data and Fisher’s exact test for dichotomous and categorical data. Missing data was assessed in each variable separately and each patient contributed to results concerning variables where his or her data were non-missing. All analyses were performed using Stata statistical software (StataCorp. 2013. Stata Statistical Software: Release 13. College Station, TX: StataCorp LP). P-value less than 0.05 was considered statistically significant.

## Results

### Demographics

The study population comprised 141 patients diagnosed with a first myocardial infarction during the time period 1995–2015; among these, 47 patients also had a diagnosis of schizophrenia at the time of their first MI. The results obtained on demographics and clinical characteristics for the different populations are shown in Table 1. Patients with schizophrenia had a statistically significant higher prevalence of smoking (p: 0.03) and a lower familial predisposition for CVD (p: 0.02) compared to their matched controls.

**Table 1:** Demographics of the study population.

	Schizophrenia, n (sd)	PHC, n (sd)	P-value	Available data*
**Age, years**	53.4 (12.0)	54.1 (12.0)	0.76	(47,93)
**BMI, kg/(m^2^)**	28.9 (6.7)	27.5 (5.3)	0.24	(32,74)
	**Schizophrenia, n (%)**	**PHC, n (%)**	**P-value**	**Available data**
**Male**	32 (68.1)	65 (69.1)	1.00	(47,94)
**Smoking**			0.03	(43,89)
→ Active	31 (73.8)	45 (50.6)		
→ Never	7 (16.7)	20 (22.5)		
→ Previous	4 (9.5)	24 (27.0)		
**Diabetes**	5 (11.6)	13 (14.6)	0.79	(43,89)
**Hypercholesterolemia**	15 (35.7)	36 (40.0)	0.70	(42,90)
**Hypertension**	15 (36.6)	35 (38.9)	0.85	(41,90)
**Family predisposition for CVD**	10 (26.3)	43 (50.0)	0.02	(38,86)

PHC, psychiatric healthy control

* number of available patients in the group of patients with schizophrenia and PHC, respectively. The values indicate the number of susceptible patients minus the number of patients with missing data because of incomplete patient journals.

### Examination and treatment

Data on three patients with schizophrenia were partly missing because of incomplete patient journals. The first patient was lost after stage 1, while two other patients were lost after stage 3. None of the PHCs had incomplete journals. The primary analysis accounted only patients with complete datasets.

Patients with schizophrenia were statistically significantly less likely to be offered and accept examination and at the same time be offered and accept treatment as compared to PHCs (p<0.01) as shown in Table 2. However, when analysing each stage individually, none of the secondary results were statistically significant (Table 2), though, a trend was found for the difference in the proportions of accepted examinations (p: 0.10) as well as accepted treatments (p: 0.09) between the two groups with a 100% acceptance among controls for both outcomes.

**Table 2:** Results on examination and treatment. Primary results show the fraction of all patients who completed all four stages of the pretreatment process with positive outcome. Secondary results show the fraction of susceptible patients who completed each stage of the pretreatment process with positive outcome. We considered a patient susceptible for a stage in the pretreatment process if the patient had completed the previous stage with positive outcome.

	Schizophrenia, n (%)	PHC, n (%)	P-value	Available data*
***Primary analysis***				
**Pretreatment completed with positive outcome**	37 (84.1)	92 (97.9)	<0.01	(44,94)
***Secondary analyses***				
**Examination was offered** (stage 1)	45 (95.7)	92 (97.9)	0.60	(47,94)
**Examination was accepted** (stage 2)	42 (95.5)	92 (100.0)	0.10	(44,92)
**Treatment was offered** (stage 3)	41 (97.6)	92 (100.0)	0.31	(42,92)
**Treatment was accepted** (stage 4)	37 (94.9)	92 (100.0)	0.09	(39,92)

PHC, psychiatric healthy control

* number of available patients in the group of patients with schizophrenia and PHC, respectively. The values indicate the number of susceptible patients minus the number of patients with missing data because of incomplete patient journals.

The types of examination offered and its distribution were as following: CAG (schiz: 91.1%, PHC: 97.9%) CT-CAG (schiz: 0, PHC: 1.1%) and exercise-ECG (schiz: 8.9%, PHC: 1.1%). CAG was the predominant type of examination offered to both populations, with higher occurrence among the controls, however, more patients with schizophrenia were offered exercise-ECG, this difference was of statistical significance (p: 0.04). Similarly, the types of treatment offered and the distribution were: medication (schiz: 14.6%, PHC: 15.4%), PCI (schiz: 82.9%, PHC: 75.8) and CABG (schiz: 2.4%, PHC: 8.8%), however, this difference in type distribution was not statistical significant.

## Discussion

Despite each step in the four-step processes being of no statistical significance when analysed individually, they as a whole contribute to the primary outcome of less cardiac treatment received in the schizophrenia population. Therefore, it seems that each step from initial admission to final decision contributed to the lower rate of treatment in the schizophrenia population. However, without statistical significance we are unable to pinpoint, whether Physician bias, patient’s unwillingness to receive health care or both contribute to the excess mortality seen in patients with Schizophrenia and first MI. Apart from the primary outcome being of significant difference, we also found a dissimilarity in the type of procedures offered between the populations. Patients with schizophrenia were less likely to be offered invasive CAG and received more offers of exercise-ECG, which was in contrast to the controls who predominantly received offers of CAG. This is in accordance to previous studies that have concluded patients with schizophrenia to have a lower rate of invasive cardiac procedures performed, amongst these, CAG [23–26]. However, for treatments, we did not find any statistically significant difference between the populations.

The difference in the offered cardiac procedures, although of no statistical significance, could possibly be explained by either the severity of MI and/or physician bias. The severity of MI was not accounted for in our study but was however concluded to be equal in a population with schizophrenia compared to the general population in a previous study [27]. Physician bias originates in the stigma around mentally ill patients, not only seen in the community but also in the hospital settings [20]. It can be either intentional or unintentional and may result in a failure to act [28]. When applied to our study this implies a failure to offer treatment to patients despite the necessity for it. The clinical manifestation of schizophrenia may be seen as a complication for postoperative care and influence final decisions of cardiac procedures following a MI. Thus, these decisions may be based on tacit assumptions rather than standard guidelines based on medical outcomes. Three of the patients in our study population with schizophrenia reported that they had previously visited the hospital complaining of typical chest pain but where sent home without examination. This is further strengthened by one study that found that patients with schizophrenia only receive care when their symptoms become severe enough [29] and that doctors tend to have a more negative attitude towards comorbid schizophrenia patients [30].

We also found a statistically trend of patients with schizophrenia being more likely to decline offers of both examination and treatment compared to the general population. This trend may be owed to the patients unwillingness to cooperate [31] or lack of insight to their illness and consequently failure to understand the importance of either being examined or treated [32,33]. This is supported by the reasons to which the patients with schizophrenia in our study declined offers. Some patients reported they did not ‘need’ the examination or treatment while others failed to turn up for the appointment, which was also considered a decline. The failure to turn up can be owed to the negative symptoms patients with schizophrenia can experience, which amongst other symptoms includes lack of interest and motivation to pursue a goal [34,35]. Furthermore, we found no difference in offers of examination and the distribution of different treatment types. These contradictive results may be explained by the small population size in our study.

This study also investigated other characteristics of the study population (Table 1) to get a general idea of whether patients with schizophrenia were somatically sicker than their controls. We expected to find a correlation, which could help explain the hypothesised fewer offers being made to the population with schizophrenia. However, we only found two significant differences between the populations; patients with schizophrenia were more likely to be smokers and had a lower familial predisposition to CVD. The higher tendency of smoking in patients with schizophrenia has previously been established [36]. Moreover, we assume that the low familial predisposition to CVD is owed to the patient’s unawareness of family medical history and has thus been reported wrongfully and not accounted for as a risk factor. However, patients with schizophrenia may also have higher risk of MI due to smoking and, consequently, familial CVD may be less relevant to them. Furthermore, patients with schizophrenia were less likely to be in treatment for diabetes, hypercholesterolemia and hypertension at the time of a first MI. This difference did not reach statistical significance but is in accordance with the CATIE study [37] that concluded low treatment for diabetes, hypercholestemia and hypertension in a population with schizophrenia. The results found in our study points to under-treatment of the patients with schizophrenia since many studies have concluded these patients to have an increased blood glucose level, a higher prevalence of diabetes type 2 [38–40] and hypercholesterolemia [39] compared to the general population.

Despite the differences in each step of the pre-treatment procedure not being statistically significant, our data show a clear difference between the two populations in healthcare and that the doctor-patient interaction may help explain the trend of fewer cardiac procedures offered and received by the patients with schizophrenia, confirming our main hypothesis.

### Clinical application

Patients with schizophrenia belong to a vulnerable group prone to comorbidity that requires special attention. In order to decrease the gap in excess mortality some changes should be implemented in the healthcare system upon initial admission. Firstly, there is a mismatch between the somatic and mental healthcare that needs to be addressed. A more integrated approach and improved collaboration between these two fields in medicine could result in earlier detection of disease that in turn would lead to better prognosis. Secondly, the handling of the patient with schizophrenia by the treating doctor needs to be reformed. It is important that the health care provider is aware of the limits of dealing with a double diagnosed patient, it also important to educate health care personnel on the issue of unintentional bias that may occur. A more personalized approach towards the patient would perhaps make the patient more susceptible to the health care system and more willing to cooperate. Ultimately, routine check-ups along with better guidance to a healthier lifestyle could decrease the incidence of CVD in the schizophrenia population.

## Conclusion

The results in the present study suggests that there is a significant difference in the proportion of patients with schizophrenia who are being offered and accepts examination and at the same time being offered and accepts treatment in comparison to a population of psychiatric healthy controls. However, we cannot pinpoint at which stage in the pretreatment process this discrepancy emerges, as all stages contribute a little, though the results of the analyses concerning the individual stages are statistically insignificant. Furthermore, the study revealed a trend towards patients with schizophrenia being more likely to decline offers made by the treating doctor for both examination and treatment. However, we suspect that the reason for these differences is a combination of physician bias and the patient’s willingness to cooperate and understand the extent of their somatic disease, where the latter plays a larger role. A more integrated somatic and mental hospital system and routine screenings could contribute to reduce the excess mortality from MI in patients with schizophrenia.

## Limitations

Greater statistical significance could have been reached with a larger sample population. However, due to the study being limited to the North Denmark Region, no more patients were available. Furthermore, we did not match our population for social class, which may have affected our results, though healthcare in Denmark is free and equally available for all permanent residents of Denmark.
